# Statistics in Dutch policy debates on health and healthcare

**DOI:** 10.1186/s12961-019-0461-y

**Published:** 2019-06-03

**Authors:** Reinie G. Gerrits, Michael J. van den Berg, Niek S. Klazinga, Dionne S. Kringos

**Affiliations:** Department of Public Health, Amsterdam UMC, University of Amsterdam, Amsterdam Public Health Research Institute, 22660, 1100 DD Amsterdam, The Netherlands

**Keywords:** Health statistics, quantification, evidence-based policy, health policy

## Abstract

**Background:**

The notion of ‘fact-free politics’ is debated in Europe and the United States of America and has particular relevance for the use of evidence to underpin health and healthcare policies. To better understand how evidence on health and healthcare is used in the national policy-making process in the Netherlands, we explore how different statistics are used in various policy debates on health and healthcare in the Dutch government and parliament.

**Methods:**

We chose eight ongoing policy debates as case studies representing the subject categories of morbidity, lifestyle, healthcare expenditure and healthcare outcomes, including (1) breast cancer screening rates, prevalence and incidence, (2) dementia prevalence and incidence, (3) prevalence of alcohol use by pregnant women, (4) mobility and school sports participation in children, (5) costs of smoking, (6) Dutch national healthcare expenditure, (7) hospital mortality rates, and (8) bedsore prevalence. Using selected keywords for each policy debate case, we performed a document search to identify documentation of the debates (2014–2016) on the websites of the Dutch government and parliament. We retrieved 163 documents and examined the policy debate cases through a content analyses approach.

**Results:**

Sources of the statistics used in policy debates were primarily government funded. We identified two distinct functions, i.e. rhetorical and managerial use of statistics. The function of the debate is rhetorical when the specific statistic is used for agenda-setting or to convince the reader of the importance of a topic. The function of the debate is managerial when statistics determine planning, monitoring or evaluation of policy. When evaluating a specific policy, applied statistics were mostly the result of routine or standardised data collection. When policy-makers use statistics for a managerial function, the policy debate mirrors terms derived from scientific debates.

**Conclusion:**

While statistics used for rhetorical functions do not seem to invite critical reflection, when the function of the debate is managerial, i.e. to plan, monitor or evaluate healthcare, their construction does receive attention. Considering the current role of statistics in rhetorical and managerial debates, there is a need to be cautious of too much leniency towards the technocratic process in exchange for the democratic debate.

**Electronic supplementary material:**

The online version of this article (10.1186/s12961-019-0461-y) contains supplementary material, which is available to authorized users.

## Introduction

There is an ongoing debate about the dividing line between facts and opinions, and the role of scientific knowledge in policy-making [1]. Terms such as ‘fact-free politics’ [2], ‘science as an opinion’ [3] and, more recently, ‘alternative facts’ [4] reveal a concern for the position and credibility of facts in both politics and policy-making.

Although the use of evidence in policy-making remains controversial, recent developments in the political landscape in Europe and the United States of America have fuelled a growing concern among scientists and others who advocate the use of evidence in policy [5]; this is illustrated by the March for Science movement [6], the Sense About Science campaign [7], and the Alliance for Useful Evidence network [8]. The discussion on the use of facts applies to all fields of policy but, in particular, to the field of health and healthcare, which has long been bound to the tradition of evidence-based practice and policy [9].

In the Netherlands, healthcare policy aims to support the delivery of high quality, accessible and affordable healthcare services to improve the health of the Dutch population [10]. As in clinical practice, healthcare policy is increasingly expected to be based on evidence [11–13]. Evidence can be used for different functions within the policy cycle, e.g. agenda-setting (where evidence is used to underpin the need for policy); policy formulation (where evidence is used as a basis for policy development); implementation (where evidence is used to determine how policy can best be materialised); and monitoring and evaluation (where the (un)intended effects of implemented policies are measured, informing the need for improvement of policy and practice) [14, 15]. However, policy-making is not a cyclical process fluently flowing from evidence to application and to full implementation. It is iterative and context bound, involving the consideration of many values of which evidence is one, along with ideology, practicability, the complexity of the subject, timeliness and the distribution of power in politics [16–18].

Statistics (quantitative information) on health and healthcare constitute an important base of evidence for health policy [19–21]. The construction of statistics requires social and intellectual investment that is often taken for granted [22]. The government has made large investments in the development and maintenance of a data infrastructure comprising registries, survey research and the development of statistics resulting from these various data sources [23]. Nevertheless, the users of statistics may show little interest in how the statistics were constructed and/or how the underlying data were collected [22].

Espeland et al. [22] describe how statistics foster cooperation and control in complex systems. Statistics enable policy-makers to evaluate healthcare and enforce sanctions or incentives, since statistics are, seemingly, easy to interpret. If they carry authority, statistics can be used ‘to persuade’. However, that authority depends on trust in the statistics’ accuracy and validity, their usefulness in solving problems, how they link those who use the statistics and those who have invested in their development, and how statistics are considered to be objective, as opposed to human judgment [22].

Consequently, to understand how statistics on health and healthcare are used in the national policy-making process in the Netherlands, insight is needed into how the purpose for which statistics are used are connected with the function of the debate, and with the sources and construction of statistics in policy debates on health and healthcare [24–26]. Such insight should increase awareness among researchers on how their research, as expressed in statistics, is used in policy debates in government and parliament. We therefore explore how different types of statistics are used in various ongoing policy debates on health and healthcare. In the Netherlands, most of the policy debates in government and parliament are documented and published (in written text). This allows systematic analysis of the use of statistics in parliamentary healthcare debates.

## Methods

### Sampling

Based on analysis of the literature and our knowledge of ongoing policy debates in health and healthcare in the Netherlands, we focused on four categories of statistics, namely (1) morbidity statistics, (2) lifestyle statistics, (3) healthcare expenditure data, and (4) statistics on healthcare outcomes. For each category, the use of statistics was analysed in two policy debates on different topics (Table 1).

**Table 1:** Policy debate cases selected for the present study

Category	Case 1	Case 2
Morbidity statistics	Breast cancer screening rates, prevalence and incidence	Dementia prevalence and incidence
Lifestyle statistics	Prevalence of alcohol use by pregnant women	Mobility and school sports participation in children
Healthcare expenditure data	Costs of smoking	Dutch national healthcare expenditure
Statistics on healthcare outcomes	Hospital mortality rates	Bedsore prevalence

In the Netherlands, although an important part of policy-making takes place at the municipality level, the present study focused solely on the national policy debate. This policy debate on health and healthcare is understood as the formal communications between government and parliament. All communication between government and parliament is documented and made public on their respective websites, including the minutes of parliamentary debate. These texts reflect the policy debate and are part of the policy context [27–30]. Consequently, with a considerable part of the policy debate on health and healthcare being published, analysis of these documents provides insight into how statistics are used in the policy process.

For each of the four categories of statistics, through purposeful sampling, we selected two policy debate cases that encompassed the formal ongoing discussions on a health topic over a 2-year period (2014–2016) [31]. The aim was to include policy debates on statistics that used different methods of data collection to underpin statistics and were subject to current policy debates at the national level. To minimise researcher bias in the selection of cases, the policy debate cases on the use of statistics in policy debates were reviewed, discussed and agreed upon by all authors.

Eight cases were chosen to represent the categories of morbidity, lifestyle, healthcare expenditure and healthcare outcomes (Table 1).

#### Morbidity

Breast cancer screening rates, prevalence and incidence were selected since these statistics are derived from routine data collection through a national cancer registry. Dementia prevalence and incidence was selected because this figure is not measured through standardised data collection but constructed through modelling.

#### Lifestyle

Prevalence of alcohol use among pregnant women was included since the statistic is derived from a single published study. Mobility rates and school sports participation in children were chosen as these statistics can be derived from multiple sources.

#### Healthcare expenditure

Dutch national healthcare expenditure was selected because of its standardised data collection method. Costs of smoking was included because it is constructed through modelling.

#### Healthcare outcomes

Hospital mortality rates were selected because of their clear registration and the obligation (since 1 March 2013) for Dutch hospitals to publish mortality data. Bedsore prevalence was included because of the difficulty to establish this using standardised measurement.

### Data collection

We identified documents describing the use of statistics in policy debates and source documents through (1) the national government website (www.rijksoverheid.nl), which contains published documents from the 11 Dutch ministries [32], and (2) the parliament website (www.tweedekamer.nl), which contains all parliamentary documents, including minutes of parliamentary debates [33].

In both websites, we restricted the search period from 8 July 2014 to 8 July 2016. For each policy debate case, we carried a document search out using selected keywords. Appendix presents details on the search methods for the policy debate documents; 163 documents were retrieved for further analysis. The documents included in the analyses are listed in Additional file 1.

For this study, since the data concerned publicly available policy documents, no ethical approval was required.

### Analysis

To explore how statistics on health and healthcare are used, we used a conventional content analysis method as described by Hsieh and Shannon [34]. First, we started by immersing ourselves in the data by reading through the documents. We discussed each case in the research group to increase our understanding of the context of each debate. We then selected utterances (text fragments) in the selected documents that refer to statistics on the case. Next, we coded elements of these utterances iteratively in MaxQDA. We extracted themes and subthemes from the data. By reading through the different debate cases and the iterative coding, we constructed the main categories that frame our results. After establishing the main categories, the coding process was reiterated and refined by revisiting the text, and deductively coding the full text within these categories. We recorded observations that referred to the sources of statistics, the type of statistics, construction of the statistics and the content of the debates, and related these observations to the different functions of use.

Analyses were performed by the first author and discussed with all co-authors during all stages of each analysis.

## Results

We identified 163 documents that describe and underpin the eight policy debates. Documents included research reports (*n* = 58), transcripts of posed parliamentary questions (*n* = 18), transcripts of plenary debates (*n* = 11), letters to/from the government (*n* = 37), appendices (*n* = 11), newspaper messages (listed as input to a debate) (*n* = 4), speeches (*n* = 2), explanatory memoranda (*n* = 3), annual reports (*n* = 13), budget texts (*n* = 5), and an amendment text (*n* = 1). Table 2 presents an overview of the type of documents per policy debate case. In the debates on bedsores, hospital mortality and costs of smoking, we included documents that did not contain direct use of statistics but in which the construction of the statistics was discussed. The included research reports either described the construction of the studied statistic (20 of the 58 reports), or reported the statistic more generally without mentioning the primary source or only providing a reference (e.g. in an ‘introduction’ section).

**Table 2:** Type of documents per policy debate case

	Breast cancer	Dementia	Alcohol use by pregnant women	Mobility in children	Costs of smoking	Dutch national healthcare expenditure	Hospital mortality	Bedsores	Total
Report	8	5	2	5	4	24	7	3	58
Parliament question	2	3	1	1		10	1		18
Plenary debate		1	1	3		3	2	1	11
Letter to/from government	5	4	2	4	1	13	7	1	37
Appendix	1	2				7		1	11
News message			1			3			4
Speech		2							2
Explanatory memorandum				1		1	1		3
Annual report				2		9	2		13
Budget				2		2	1		5
Amendment								1	1
Total documents	16	17	7	18	5	72	21	7	163
Utterances included in the analyses	17	29	7	23	8	42	24	8	

### Characterisation of analysed debate and sources of statistics

#### Dementia

The policy debate on dementia focuses on the recent Deltaplan Dementia. This plan involves a programme stimulating interventions/research on dementia. The name ‘Deltaplan’ is a metaphor, referring to the major reconstruction of the Dutch Delta, indicating that the plan aims for a comprehensive change in the field of dementia care. In all the analysed documents, the prevalence figures used concern a statistic that was calculated by extrapolating a prevalence figure from a scientific publication (dating from 1996) from a neighbourhood to country level, and to the present time. While having the same origin and referring to the same sources, the figures used in these documents ranged from 230,000 to 260,000. In one document (an answer to a parliament question), a different prevalence figure (i.e. 80,000) was used by the minister, i.e. a statistic derived from a GP registration (NIVEL zorgregistraties Eerste lijn). We identified 17 documents in the debate on dementia.

#### Breast cancer

The statistics used in this policy debate were derived from the Netherlands Cancer Registry. When a statistic on breast cancer was featured in the debate, a reference was made to this registry, either through research (institutes) providing these statistics or to the website publishing the registry data. Statistics are routinely collected for this registry. The debate centres on breast cancer screening, the development of breast cancer in society and the financing of breast cancer treatment. We identified 16 documents in the debate on breast cancer.

#### Alcohol intake in pregnancy

Debate on alcohol intake in pregnancy is part of the discourse on prevention through lifestyle change. Documents indicated that both government and parliament support a change of lifestyle behaviour through policy. Statistics on alcohol use in pregnant women were derived from a study performed by two Dutch research institutes (Trimbos Institute and TNO). The statistics were based on survey research, which was repeated in 2007, 2010 and 2014. We identified 7 documents in the debate on alcohol intake in pregnancy.

#### Mobility in children

The debate on mobility in children is also part of the discourse on prevention through lifestyle change. This debate focuses on two objectives, namely the participation in sports by children and sports education in schools. The debate centres on the role of government and possible policy to increase exercise in children. Statistics used in this debate are derived from three sources, namely the Health Behaviour in School-aged Children study (http://www.hbsc.org/), the Lifestyle Monitor (https://www.rivm.nl/leefstijlmonitor) and the Health Survey (https://www.monitorgezondheid.nl/). For sports participation, we examined the statistics used in the debate on the number of hours of physical education in schools. Statistics used to indicate the overall exercise rates by children are derived from the OBiN study (Accidents and Exercise in the Netherlands). We identified 21 documents in the debate on mobility in children.

#### Healthcare expenditure

Healthcare expenditure includes expenditure as part of the governmental budget, as a total of expenditure for different healthcare sectors, and as total expenditure development in healthcare expenditure over the years. Statistics used are provided by Statistics Netherlands. The current debate focuses on the national policy to get a grip on health spending and to make the system more economically sustainable. We identified 72 documents in the debate on healthcare expenditure.

#### Societal costs of smoking

Statistics on the societal costs of smoking provide a financial perspective on smoking in society. However, ‘costs of smoking’ does not have one commonly accepted definition. Statistics are derived from different reports/sources that either state that the societal costs of smoking are high, or that smoking does not result in increased costs for society. No statistics on the costs of smoking were used directly in the debate. To focus attention on the harmful effects of smoking, in a few supporting reports, the high costs of smoking for society were mentioned but without substantiating the argument with statistics. We identified five documents in the debate on societal costs of smoking.

#### Bedsores

The current debate on the rate of bedsores centres on the high prevalence of bedsores in the Netherlands compared to other countries in Europe, highlighting the need to decrease this rate. All statistics used in this debate are derived from the National Prevalence Measurement of Care problems 2013. We identified 7 documents in the debate on bedsores.

#### Hospital mortality

The current debate on hospital mortality rates focuses on the development of a standardised method of data reporting. Hospitals are obliged to publish these statistics, with the Hospital Standardized Mortality Ratio as the intended measure. Currently, the studied debate focuses on the construction of these statistics. We identified 21 documents in the debate on hospital mortality.

### Sources across cases: government and non-government related

A reference was frequently provided for the statistics that were used (99 of 174 statistics). These sources concerned reports (*n* = 65) (including reports that were part of our sample, *n* = 20), websites (*n* = 16), scientific studies (*n* = 7), news articles (*n* = 2) and others (*n* = 9).

Of the 99 referenced sources, 74 concerned governmental agencies (e.g. National Institute for Public Health and the Environment (RIVM), The Netherlands Institute for Social Research, Statistics Netherlands) or organisations funded by the government to conduct research on the respective topic. Specifically, governmental agencies (*n* = 41), consultancies (*n* = 16) and research institutes (*n* = 17).

### Type of statistics across cases, and use of tables and visualisations

The identified datasets used both rounded (*n* = 86) and exact statistics (*n* = 53). Rounded statistics are those that can be rounded up or down, mentioned in words, or expressed through normative words (e.g. ‘strongly decreased’). Exact statistics are precise to a single digit in absolute form, or expressed in exact percentages. In all debates, statistics were used as rounded statistics at least once. In the debate on dementia, the prevalence figure was presented only as a rounded figure, and always including terms such as ‘more than’ or ‘approximately’.

Visualisation was used to present statistics in the debates on healthcare expenditure (*n* = 7), mobility in children (*n* = 7), breast cancer (*n* = 2), hospital mortality (*n* = 5) and bedsores (*n* = 1). These visualisations were used in reports. Additionally, tables were frequently used to present statistics on healthcare expenditure (*n* = 13).

#### Example of a rounded figure

**Healthcare expenditure:** “*Healthcare expenditure is the largest government expenditure after social security. In the Netherlands, we collectively spent around €71 billion on healthcare in 2015, about 11% of our GDP*.” (Answer to parliament question, 3 February 2015).

#### Example of an exact figure

**Healthcare expenditure:** “*In 2014, collective healthcare expenditure rose by 0.1 percentage points, to 10.0% of the GDP. In 2015, the collective healthcare expenditure will decrease by 0.1 percentage points to 9.9%, equal to the level of 2012 and 2013. The nominal growth of healthcare expenditure will decrease from 2½ % in 2014 to 1¼ % in 2015.*” (Report: Macro economische verkenning 2015, by CPB, 10 September 2014).

### Functions of policy debates: rhetorical and managerial

We identified two functions for the use of statistics in the policy debates, i.e. rhetorical and managerial. First, when statistics are used to convince the listener to act, the function of the debate is rhetorical; the actual number does not affect its use in the debate, but seems to indicate ‘a lot’. Second, statistics are used for a managerial function when planning, monitoring or evaluating specific healthcare policy. When used managerially, the number itself is instrumental to the decisions made in planning, monitoring or evaluating specific policy; when the number changes, so does the decision to be taken.

In the studied policy debates, when statistics were used for a managerial function, the way in which the statistics were constructed became a topic of discussion. In addition, when statistics were used to evaluate the effectiveness of a particular policy, the policy-makers adopted the terms used by the research community.

#### Use of statistics for rhetorical functions

When used rhetorically, policy-makers used statistics as argumentation tools to recruit support and to place or maintain issues on the policy agenda. When used rhetorically in the studied documents, statistics were mostly rounded and the exact number did not seem to be relevant to the discussion.

For example, in the debate on dementia, the prevalence figure was used to emphasise the problem of dementia in society.

**Dementia:** “*Care for people with dementia is high on the societal and political agenda. Research indicates that the number of people with dementia will increase sharply in the coming years. It is expected that by 2040 half a million Dutch people will have a form of dementia. At the moment that is half* [of half a million].” (Report: ‘Kijken met andere ogen naar zorg voor mensen met dementia en onbegrepen gedrag, by the Ministry of Health Welfare and Sports, June 2015)

Another example is the debate on prevalence of breast cancer. In the following quote from a report on the reimbursement of cancer treatment, the size of the problem of breast cancer in society was illustrated through a rounded statistic. Only after this rhetorical introduction, the specifics of the reimbursement of the treatment were explained. No references were provided for the incidence rate of 14,000.

**Breast cancer**: “*Every year, 14,000 women and 100 men are diagnosed with invasive breast cancer in the Netherlands. More and more women are using the possibility of a breast reconstruction after a breast removal operation to treat breast cancer*.” (Report: Voorwaardelijke toelating tot het basispakket, Voortgangsrapportage, by the National Health Care Institute, 21 June 2016)

In addition, if the statistic is used primarily to persuade, the statistic is used rhetorically. In the debates on breast cancer, healthcare expenditure, mobility in children, bedsores and alcohol use among pregnant women, the statistics were used to evaluate policy direction.

Two examples of this:

**Mobility in children:** “*Too many young people exercise too little. Less than half of the Dutch youngsters does not adhere to the norm that was chosen as a baseline for policy to stay healthy and fit.*” (Explanatory memorandum to a proposed bill, 25 February 2016)

**Alcohol use in pregnant women:** “*How do you explain that pregnant women who drink alcohol have started drinking more?*” (Parliament question, 20 August 2015)

The minister addressed this rhetorical question with an exact, managerial, answer:

“*With this correspondence, I am informing you of the manner in which the evaluation of the alcohol Licensing and Catering act will be executed.* [ … ]. *In 2007, 2010 and 2015 the TNO* [Netherlands Organisation for Applied Scientific Research] *carried out national polls in which, amongst others, it was asked how many women used alcohol during pregnancy and breastfeeding* [ … ]. *The new statistics show a decrease in the percentage of women drinking alcohol in the first three months of pregnancy as compared to 2010 and 2007 among all education levels: from 16.5 percent in 2007, 13.8 percent in 2010, to 6.9 percent in 2015.* [ … ] *this makes me feel optimistic.*” (Letter from the government, 1 February 2016).

### Use of statistics for managerial functions

Statistics are used for a managerial function when the figure itself is instrumental in the decision. For instance, in the debate on mobility in children, the percentage of children that received the recommended amount of physical education was used to argue for a specific policy, i.e. a mandatory number of hours of physical education in schools. The statistic itself is what determines the decision, as the percentage of children that received physical education was considered to be too low by the opposition.

When used managerially, statistics are most likely to be exact; however, there are some exceptions to this rule. In the debate on dementia, a rounded statistic was used managerially when it was instrumental in determining the amount invested in Deltaplan Dementia. In the debate on mobility in children, both rounded and exact statistics were used interchangeably.

Some statistics are intended to be used managerially by the policy-maker such as the bedsores statistics and hospital mortality rate. However, in the studied documents, the application of these statistics for practical decisions was rejected. In an exchange between government and parliament, a member of parliament proposed to introduce a financial reward for those who keep the bedsore rates below a certain benchmark; however, the statistics were not considered sufficiently reliable by the minister to implement this idea (see quote).

**Bedsores:** “*For those healthcare providers who for, at most, three percent of the total number of insured to whom care is given suffer from bedsores, malnutrition, or dehydration, receive from our minister a modest bonus for the purpose of the workplace.*” (Amendment proposal, rejected, 15 September 2014)

Outside government policy, the hospital mortality statistic is used for ‘internal quality improvement in hospitals’. The government stimulates improving the transparency and credibility of the statistics. In the studied texts, the managerial use of hospital mortality statistics to drive policy action was discussed. In an intended managerial use of a mortality figure, a higher risk of mortality in the weekend was used to request the government to act. Nevertheless, the debate does not prompt action, but focuses on the credibility of the figure. This discussion is illustrated in the following section.

### Scientific discussion and managerial use of statistics

The need for scientific research is repeatedly mentioned in the different debates. Policy-makers discussed the reliability and credibility of statistics, especially when the statistic was used managerially. In the studied debates, when used rhetorically, the construction of the statistics was not questioned.

In the debate on hospital mortality and bedsores, policy-makers did not consider the statistics to be solid enough for decision-making. Here, they adopted arguments used by the scientific community (e.g. methodological criticism on the construction of the figure). The discussion on the statistic itself was illustrated by a question posed in parliament on higher hospital mortality during the weekends. The statistic was used to identify a possible healthcare problem, namely that of higher mortality in hospitals during the weekend, implying that care during the weekend might be substandard. The Minister of Health addressed the issue by providing an explanation on how the statistic was constructed, using argumentation provided by the scientific community on case-mix adjustment (adjusting to a differing mix of patients with regard to illness severity) to explain that statistic. Consequently, the discussion focused on the uncertainty of what the statistic indicates, rather than how to address possible problems related to the quality of hospital care during the weekends.

The quote below illustrates how a parliament member asked a question about higher hospital mortality on the weekend. The answer by the Minister refers to published research, explaining that a ‘case mix’ causes this statistic to be higher. Thus, the Minister used the uncertainty of the methodology as an argument not to address the issue at hand.

**Hospital mortality:** “*Do you share the opinion that a 20% higher mortality risk in the weekends is so shocking that something needs to be done about this immediately? If yes, what do you propose?*” Parliament member.

*From the* [Monitor adverse events in Dutch hospitals 2011/2012]*, that EMGO/NIVEL carried out on my request* [ … ]. “*A possible explanation for this – against the background of the risk of healthcare-related damage found – is that the so-called ‘case mix’ of patients admitted on the weekend means that these patients are, on average, sicker.*” (Minister of Health, Parliament question, 2 September 2015)

In the debate on dementia, the prevalence figure was used managerially as a basis for the evaluation of Deltaplan Dementia. In the quote below, two statistics are compared to clarify the use of the figure. Nevertheless, as opposed to other statistics that are used managerially, this statistic is neither exact nor the result of routine data collection, but is constructed through modelling, representing an exception to our observations regarding the use of exact statistics related to the managerial use of statistics.

**Dementia:** “*The estimation of 230,000 to 250,000 people with dementia in the Netherlands, on which the analysis of the Deltaplan Dementia is also based, is somewhat similar to international studies providing statistics for Western Europe. The RIVM bases the statistic of 80,000 on a sample from the GP registration database. The RIVM, however, mentioned that this does not provide a complete overview because it concerns a sample and also because GPs lack a complete registration of people with dementia.*” (Answer to parliament question by the government, 17 March 2015)

### Evaluation of specific policy through the use of statistics

In the debates on breast cancer, healthcare expenditure, mobility in children and bedsores, statistics were used to managerially evaluate the effectiveness of the policies set by the government. We consider the use of statistics as a ‘managerial evaluation’ when the research was conducted with the explicit aim to evaluate a specific policy. These evaluations are intended to inform the managerial use of statistics.

For example, statistics on breast cancer incidence and prevalence are used to evaluate established programmes and treatment of breast cancer. As such, the use of the screening programme was evaluated and confirmed to be effective.

**Breast cancer:** “*The conclusion is that the population screening yields considerable health benefits and the Health Council recommends continuing and further improving population screening.* [ … ] *A total of 6,975 cases of breast cancer have been detected and the detection rate has increased to 6.9 per 1000 women tested.*” (Appendix to a letter to parliament, 2016)

#### Construction of the statistics and evaluation of specific policy

The type of data collection and evaluation of specific policy appears to be connected. All statistics used for managerial evaluation of policy were constructed through routine or standardised data collection. Statistics on national healthcare expenditure, mobility in children and breast cancer were either constructed through standardised or routine data collection.

The statistics on dementia, costs of smoking and alcohol use among pregnant women were not used for specific policy evaluation. It seems that no defined method of constructing statistics on the costs of smoking was determined, and no effort was made on a policy level to strengthen these statistics. In the debate on alcohol use among women, measurements were conducted through questionnaires and, since 2007, have been repeated twice. The methodology behind these statistics is not questioned when used; however, they are not used for the evaluation of a specific policy.

In the studied debates, the discussion on the methodology behind bedsores and hospital mortality statistics was taken up in the policy debates with a managerial function. Nevertheless, the policy-makers using these statistics did not consider them to be adequate to be used for implementation and evaluation.

## Discussion

We explored how different types of statistics are used in a variety of ongoing policy debates on health and healthcare. Statistics used were mostly derived from research directly commissioned by the government, or produced by government organisations. The main sources of the statistics were reports, websites and (occasionally) scientific studies. We distinguished two distinct functions of policy debate – rhetorical and managerial use of statistics. If the debate’s function is rhetorical, statistics were used as an argumentation tool to show the success of (or need for) a certain policy, to recruit support and to place/maintain issues on the policy agenda. The debate’s function is managerial when statistics were used to determine how specific policy measures are planned, monitored or evaluated. Statistics that are used managerially are primarily exact, expressed in tables or visualised, while rounded statistics are primarily used for rhetorical functions. When evaluating specific policy, statistics are exclusively the result of routine or standardised data collection. Furthermore, when statistics are used (or intended) for a managerial function, the debate within policy mirrors the debate on the construction of these statistics in the scientific community.

### Limitations

We explored the use of statistics in the Dutch debate on health and healthcare based on eight case studies. The debate on health and healthcare consists of numerous types of statistics and topics. A large part of the included documents was connected to one case, i.e. healthcare expenditure (*n* = 72), while other cases included fewer documents, e.g. alcohol use by pregnant women (*n* = 7) and costs of smoking (*n* = 5). The larger number of documents within the healthcare expenditure case allowed for a broader comparison of managerial and rhetorical use of these statistics within the same context. Nevertheless, the insights gained were not dominated by one single case (i.e. healthcare expenditure), as valuable insights of the use of statistics were retrieved throughout all cases by observing the use of statistics in different contexts. A wider selection of cases would have resulted in a more comprehensive insight into how statistics are used across different topics. Simultaneously, an analysis of each single policy debate case and even every single text would provide a deeper understanding of how these statistics are used in their respective contexts.

The texts involved in the policy debate were derived from two websites; however, it is likely that not all documents published on these websites are represented in this study. Nevertheless, based on the single search terms used, a large part of the discourse was identified. Additionally, policy debate in the Netherlands encompasses many actors outside a governmental setting. We decided to focus on debates around the policy processes in government and parliament. Extending the analysis to include more actors would have resulted in extended insight into their influence on the of statistics in policy debate.

Our distinction between rhetorical and managerial functions is based on our interpretation of the documents and its contents. Nevertheless, these functions may overlap, e.g. when a parliament member proposes a law to make a statement (i.e. a rhetorical function) rather than to achieve a change in regulation (i.e. a managerial function).

Our results represent only those cases that were studied and cannot be generalised to all debates on healthcare involving the use of statistics in the Dutch government and parliament. Nevertheless, our results provide insight into how statistics are used in these debates and what might be expected in others.

### Interpretation

Most of the sources included in the policy debate were related to the government, as also found by an earlier study on ex ante policy studies in the Netherlands [35]. This coproduction of research and policy is considered to be an essential part of evidence-based policy in the Netherlands [36].

In the studied documents, policy-makers used arguments in which the scientific terms ‘case mix’ and ‘significance’ were mentioned, mirroring the language used in scientific debates. By using scientific language, the policy-makers and scientific institutions may gain more authority over the policy process. However, scientific language may also be a means to shift attention from societal problems to scientific/methodological problems. Then, methodological arguments could be used to discredit the evidence if it does not align with a certain political agenda. Conversely, if a statistic is trusted to be valid and accurate [22], the actual problem at hand might be discussed rather than the construction of the statistic itself.

In the debates, only a few scientific publications were used as a source. The statistics used were published in reports or derived from websites. It seems that the usability of statistics for the evaluation of policy increases when data are collected routinely or through standardised methods. To embed statistics in the managerial policy debate, it appears worthwhile to invest in routine or standardised measurements. The results of this study support Cairney and Oliver [37], who proposed to reduce the uncertainty of research results, increasing the validity and reliability of statistics to encourage the managerial use of evidence in policy.

Further research on evidence-based policy could focus on the use of statistics in policy debate in other countries. It would be interesting to establish whether the connection between the managerial use of statistics, scientific discussion and the routine data collection of statistics is similar to those in the debates we studied in the Netherlands. Moreover, the relation between the current debates and change in debates over different time periods might provide useful insight into the managerial and rhetorical use of statistics over time. Additionally, to improve understanding of the role of managerial and rhetorical use of statistics in the decision-making process, future research could address how these functions are applied to guide choices between particular policy interventions.

#### Implications for policy and practice

It has been argued that policy-makers lack the knowledge and time to critically evaluate the statistics they apply [38, 39]. Statistics might be used in contexts and for purposes other than that for which they were initially created [40–42]. The results of this study indicate that policy-makers do take the time to understand the construction of statistics and refer to scientific discussions when the statistic is intended to be used managerially. Questions are asked on the statistics used to shape policy; therefore, policy-makers might need to relate the construction of the statistics to defend their policy choices and might need to prepare for that discussion. While this attention to the construction of statistics seems positive for evidence-based policy, the use of statistics to lend objectivity to policy decisions has a darker side. Currently, research is focused on ‘getting more evidence into policy’, rather than on the suitability of evidence in the policy process [43, 44]. If healthcare policy is increasingly based on research alone, the debate on what policy may be implemented would be led by the scientific community rather than the judgment of policy-makers. Consequently, policy decision-making may become a purely technocratic process, rather than a democratic one [45].

Moreover, the number of government-related sources used indicates that the statistics applied in the policy are mainly government driven. While this co-production of evidence is considered a strong feature of Dutch policy-making and could support effective implementation of evidence in policy [45], government influence might also affect the outcomes of research. Researchers should be aware that the statistics used by policy-makers are primarily derived from government-related institutions and routine or standardised data collection. As the government may invest in research on topics that they are interested in, those topics that do not have government priority might become under-investigated [46]. Moreover, reports funded by the government could be skewed to display a positive view of government action or policy [35, 47]. Consequently, with much of the evidence used in policy being government related, researchers need to continue to be transparent concerning their methods and the nature of government involvement.

## Conclusion

Our results indicate a rational process of integration of statistics as evidence in the policy process when used for decision-making. Whereas the statistics used for rhetorical functions do not seem to invite critical reflection, when the goal of the debate is managerial, i.e. to plan, monitor or evaluate healthcare, the construction of the statistics receives consideration by parliament. Considering the current role of statistics as a rhetorical and managerial argumentation tool, there is a need to be cautious of too much reliance on statistics for all policy decisions in exchange for a balanced democratic debate in evidence-based policy-making.

### Additional file

Additional file 1: Documents included in the analyses. (DOCX 22 kb)

## Data Availability

All documents analysed during this study are included in this published article and Additional file 1.

## Appendix

### Document identification

Documents were identified through two websites: www.rijksoverheid.nl and www.tweedekamer.nl. The former uses a search function that finds only the exact words entered in the search bar, while the latter finds the stem of the words entered. Search results were assessed by title for relevance (excluding documents which had no relation to the topic). No documents were excluded based on the title alone. All other documents were downloaded and examined for the use of statistics. First, in the table of contents, chapters that referred to the topic were identified and read. Second, keywords were used to search for the topic within the documents. Search terms regarding the case were applied, using the stem of the topic (e.g. for the case ‘mobility in children’ a search was carried out using the terms “*beweg*”/“*beweeg*” and “*kind*”). If an abbreviation was used within the text (e.g. “BK” for “breast cancer”), this term was additionally applied in the search function. The page containing the word was read, whilst looking for the use of statistics. Documents with less than 10 pages were read in their entirety. Documents in which statistics were used regarding the particular case were included. After inclusion of the documents for analysis, overlapping documents were removed. In addition, we included four reports referenced in the parliament questions that were necessary to understand the statistics used in the debates. In total, 163 documents were included in the analysis. The number of documents identified per search is described in Table 3.

**Table 3:** Document search methods

		www.rijksoverheid.nl		www.tweedekamer.nl		External source	All
Case	Search terms (in Dutch)	*n*	*n* included	*n*	*n* included	*n* included	Total *n* included
Dementia	dementie	322	12	298	12	1	17
Breast cancer	borstkanker	77	9	64	9	1	16
Alcohol use by pregnant women	zwanger alcohol	15	4	16	4	1	7
	zwanger drank	5		3			
Mobility among children	bewegen kinderen leefstijl	76	11	76	9	0	18
	beweging kinderen leefstijl	58					
	beweging kind leefstijl	21					
	bewegen leefstijl kind	26					
National healthcare expenditure data	zorguitgaven	182	29	180	46	0	72
Costs of smoking	roken kosten	130	3	56	4	0	5
	roken kost	46					
	roken uitgaven	47		12			
	roken MKBA	6		4			
Bedsores	decubitus	24	3	16	4	1	7
	doorligwonden	6		9			
Hospital mortality	ziekenhuis sterfte	63	12	45	11	0	21
	ziekenhuissterfte	4		2			
	hospital standardized mortality ratio	5		8			
	HSMR	2		2			
	ziekenhuis mortaliteit	14		6			

## Abbreviations

RIVM: National Institute for Public Health and the Environment
