# Supplementary material for: Statistics in Dutch policy debates on health and healthcare
Source: Health Res Policy Syst. 2019 Jun 3;17:55. doi: 10.1186/s12961-019-0461-y (PMC6547579; doi:10.1186/s12961-019-0461-y)
Supplement: Supplementary file 1 — Documents included in the analyses. (DOCX 22 kb) [file 12961_2019_461_MOESM1_ESM.docx]

**Supplementary Material to ‘Statistics in Dutch policy debates on health and healthcare’**

**Included documents per policy debate case**

**Dementia**

1. Minister van Volksgezondheid Welzijn en Sport. **Beantwoording kamervragen over het bericht ‘Drankje tegen Alzheimer Souvenaid te snel op de markt’**  17-03-2015.

2. **Bijlage 1 Dementie**  Unknown.

3. **Bijlage 2: Het Deltaplan Dementie**  Unknown.

4. Staatssecretaris van Volksgezondheid Welzijn en Sport. **Brief Geestelijke gezondheidszorg Mantelzorg**  7-07-2015.

5. Staatssecretaris van Volksgezondheid Welzijn en Sport. **Brief Langdurige zorg**  26-02-2016.

6. Staatssecretaris van Volksgezondheid Welzijn en Sport. **Brief Zorg en maatschappelijke ondersteuning**  2-05-2015.

7. E.B. Birkenhager-Gilesse M. Breteler, F. van Harskamp, I. de Koning, A. Hofman. **De prevalantie van ouderen van de ziekte van Altzheimer, vasculaire dementie en dementie bij de ziekte van Parkinson; het ERGO onderzoek**

8. RIVM. **Een gezonder Nederland**  2014.

9. Verslag van een algemeen overleg. **Eerstelijnszorg**  22-09-2014.

10. Staatssecretaris van Volksgezondheid Welzijn en Sport. **Kamerbrief sameleven met dementie**  8-07-2015.

11. NIVEL. **Kennissynthese Vrijwilligershulp thuis bij mensen met dementie**  Februari 2016.

12. IGZ. **Kijken met andere ogen naar de zorg voor mensen met dementie en onbegrepen gedrag**  Juni 2015.

13. Vragen gesteld door de leden der Kamer. **Over het bericht «Drankje tegen Alzheimer Souvenaid te snel op de markt»**  4-02-2015.

14. **Toespraak staatssecretaris Van Rijn bij EU-conferentie over dementie**  9-05-2016.

15. **Toespraak van staatssecretaris Van Rijn bij de opening van de expositie 'Gezichten van dementie'** 10-03-2016.

16. Verslag houdende een lijst van vragen en antwoord. **Vaststelling van de begrotingsstaten van het Ministerie van Volksgezondheid, Welzijn en**  13-11-2014.

17. IGZ. **Zo houdt de inspectie de komende jaren toezicht op de verpleeg(huis)zorg**  Unknown.

**Breast cancer**

1. Panteia. **Beleidsdoorlichting ziektepreventie**  Maart 2015.
2. Staatssecretaris van Volksgezondheid Welzijn en Sport. **Beleidsnota Rampenbestrijding**  7-07-2016.
3. **Bijlage 1 Stand van zaken acties en activiteiten Rijksoverheid**  Eind 2015.
4. Minister van Volksgezondheid Welzijn en Sport. **Brief van de minister van volksgezondheid, welzijn en sport over Geneesmiddelenbeleid**  7-04-2016.
5. Minister van Volksgezondheid Welzijn en Sport. **Brief van de minister van volksgezondheid, welzijn en sport over Herziening Zorgstelsel**  26-05-2015.
6. ZI. **Brief: Rapport Zinnige Zorg - Systematische Analyse Nieuwvormingen**  16-04-2016.
7. Trimbos instituut. **Depressiepreventie: gerichte aanpak voor risicogroepen**  2014.
8. IBMG Erasmus Universiteit Rotterdam;. **Groot onderhoud van de diagnosekostengroepen (DKG’s) in het risicovereveningsmodel voor de somatische zorgkosten**  16-04-2015.
9. IGZ. **Het resultaat telt ziekenhuizen 2013**  Februari 2015.
10. IGZ. **Het resultaat telt ziekenhuizen 2014**  Januari 2016.
11. Vragen gesteld door de leden der Kamer. **Hormoon verstorende stoffen en de relatie met de volksgezondheid**  08-03-2016.
12. met de daarop door de regering gegeven antwoorden Vragen gesteld door de leden der Kamer. **Hormoon verstorende stoffen en de relatie met de volksgezondheid** 20-04-2016.
13. GGD West Brabant. **Incidentie van kanker in de gemeente Moerdijk (2004-2013)**  Juni 2016.
14. ZI. **Pakketcriteria pertuzumab**  20-01-2016.
15. ZI. **Rapport Zinnige Zorg - Systematische Analyse Nieuwvormingen**  16-04-2015.
16. ZI. **Voorwaardelijke toelating tot het basispakket Voortgangsrapportage 2016**  21-06-2016.

**Alcohol use by pregnant women**

1. TNO. **Alcoholgebruik tijdens zwangerschap en borstvoeding**  Publication date unknown.
2. Erasmus MC. **Eindrapportage Aanpak babysterfte in Nederland**  Maart 2016.
3. Staatssecretaris van Volksgezondheid Welzijn en Sport. **Kamerbrief over voortgang alcohol januari 2016**  01-01-2016.
4. Staatssecretaris van Volksgezondheid Welzijn en Sport. **Vragen gesteld door de leden der Kamer, met de daarop door de regering gegeven antwoorden [bericht: Zwangere vrouw drinkt meer alcohol]**  28-09-2015.
5. Minister van Volksgezondheid Welzijn en Sport. **Zorg rond zwangerschap en geboorte**  07-03-2016.
6. Verslag van een algemeen overleg. **Zorg rond zwangerschap en geboorte.** 22-01-2015.
7. Nederlands Dagblad. **Zwangere vrouw drinkt meer alcohol**  19-08-2015.

**Mobility among children**

1. **Beantwoording begrotingsvragen Jeugd en Sport**  Date unknown.
2. Verslag van een algemeen overleg. **Bewegingsonderwijs**  24-09-2014.
3. Minister van volksgezondheid welzijn en sport. **Brief Geannoteerde agenda van de Sportraad op 25 november 2014**  18-11-2014.
4. Minister van Volksgezondheid Welzijn en Sport. **Brief van de minister van volksgezondheid, welzijn en sport over Geannoteerde agenda van de Jeugd en Sportraad**  28-04-2015.
5. Staatssecretaris van Volksgezondheid Welzijn en Sport. **Commissiebrief inzake Verzoek om reactie op het HBSCrapport 2013**  09-10-2014.
6. CBS; NIVEL; NJi; NZA; RIVM; SCP; Trimbos instituut; ZIN. **De Staat van Volksgezondheid en Zorg Kerncijfers voor beleid - een introductie -**  Mei, 2016.
7. **Geannoteerde agenda Sportraad 25 november 2014 te Brussel**
8. Universiteit Utrecht; Trimbos Insituut; SCP. **Gezondheid, welzijn en opvoeding van jongeren in Nederland HBSC 2013**
9. Verslag van een notaoverleg. **Initiatiefnota van het lid Rudmer Heerema: «Lichamelijke opvoeding is een vak, juist in het basisonderwijs: Een pleidooi voor beter bewegingsonderwijs op de basisschool gegeven door vakleerkrachten»**  23-05-2016.
10. **Plan van aanpak bewegingsonderwijs**  23-05-2015.
11. SCP. **Rapportage sport 2014**  Januari 2015.
12. Welzijn en Sport Ministerie van Volksgezondheid. **Rijksbegroting 2015 xvi Volksgezondheid, Welzijn en Sport**  16-09-2014.
13. **Rijksjaarverslag 2014 xvi Volksgezondheid, Welzijn en Sport**  20-05-2015.
14. **Rijksjaarverslag 2015 xvi Volksgezondheid, Welzijn en Sport**  18-05-2016.
15. TNO. **Trendrapport Bewegen en Gezondheid 2000/2014**  2015.
16. **Vaststelling van de begrotingsstaten van het Ministerie van Volksgezondheid, Welzijn en Sport (XVI) voor het jaar 2016**  15-09-2015.
17. Verslag van een wetgevingsoverleg. **Vaststelling van de begrotingsstaten van het Ministerie van Volksgezondheid, Welzijn en Sport (XVI) voor het jaar 2016**  22-12-2015.
18. Memorie van toelichting. **Voorstel van wet van het lid Van Nispen tot wijziging van de Wet op het primair onderwijs, de Wet op de expertisecentra en de Wet primair onderwijs BES ter invoering van regels over de kwalificatie van docenten en het vaststellen van een minimum aantal uren voor wat betreft het bewegingsonderwijs**  vergaderjaar 2015–2016.

**National health care expenditure data**

1. Algemene rekenkamer. **Achtergronddocument Bezuiniging op de zorgtoeslag Realisatie en effect**  Unknown.

2. BPSEconomics. **Allocatie professionals in de curatieve zorg**  Mei 2015.

3. **Antwoorden op Kamervragen Miljoenennota 2015**

4. Minister van Volksgezondheid Welzijn en Sport. **Antwoorden op kamervragen over hoe de kosten van het huidige zorgstelsel ongezien naar de burger verschoven**  9-02-2016.

5. CAOP. **Arbeid in Zorg en Welzijn, Jeugdzorg en Kinderopvang 2015**

6. **Beantwoording kamervragen over Eerste suppletoire begroting 2016**

7. **Beantwoording vragen aan de regering over Financieel Jaarverslag Rijk 2014**  Unknown.

8. rijksoverheid. **Beleidsdoorlichting begrotingsbeleid**  Juli 2016.

9. **Bijlage 2: Verticale toelichting 2016**

10. **Bijlage 2: Verticale Toelichting Najaarsnota 2014**

11. **Bijlage 2: Verticale Toelichting Najaarsnota 2015**

12. **Bijlage bij Het Nederlandse Stabiliteitsprogramma 2016**

13. Staatssecretaris van Volksgezondheid Welzijn en Sport. **Brief Arbeidsmarktbeleid en opleidingen zorgsector**  9-02-2016.

14. Minister van Volksgezondheid Welzijn en Sport. **Brief instelling van een Technische werkgroep Beheersinstrumentarium Zorguitgaven**  8-07-2016.

15. Staatssecretaris van Volksgezondheid Welzijn en Sport. **Brief Kwaliteit van Zorg**  19-02-2016.

16. Minister van Financien. **Brief Najaarsnota 2015**  27-11-2015.

17. Minister van financien. **Brief Nederlandse Stabiliteitsprogramma 2016**  12-04-2016.

18. Minister van Financien. **Brief Nota over de toestand van ’s Rijks Financiën**  1-07-2016.

19. Minister van Volksgezondheid Welzijn en Sport. **Brief Nota over de toestand van ’s Rijks Financiën**  6-07-2016.

20. Minister van Volksgezondheid Welzijn en Sport. **Brief Toekomstig sportbeleid**  17-09-2015.

21. Algemene rekenkamer. **Brief Toepassing van de Wet openbaarheid van bestuur**  24-03-2016.

22. Minister van Volksgezondheid Welzijn en Sport. **Brief Totstandkoming rekenpremie**  18-11-2014.

23. De Algemene Rekenkamer. **Brief Vaststelling van de begrotingsstaten van het Ministerie van Volksgezondheid, Welzijn en Sport (XVI) voor het jaar 2015**  5-11-2015.

24. Minister van financien. **Brief Voorjaarsnota 2016**  27-05-2016.

25. CPB. **Broos herstel Koopkracht stijgt Macro Economische Verkenning 2015**  September 2014.

26. NIVEL. **De aantrekkelijkheid van werken in de zorg 2015 Cijfers en trends**  2016.

27. **Evaluatie normeringsystematiek gemeentefonds en provinciefonds 2010-2015**  April 2016.

28. Andersson Elffers Felix. **Evaluatie van de Wet Marktordening Gezondheidszorg (WMG) en de Nederlandse Zorgautoriteit (NZa)**  1-09-2014.

29. KPMG. **Evaluatie Zorgverzekeringswet**  September 2014.

30. Brief van de algemene rekenkamer. **Financieel Jaarverslag van het Rijk 2014**  18-05-2016.

31. Rijksoverheid. **Financieel Jaarverslag van het Rijk 2014**  20-05-2015.

32. **Financieel jaarverslag van het Rijk 2014 lijst van vragen en antwoorden**  11-06-2015.

33. Gezondheidsraad. **Hemofilie, hiv en de Wbmv**  21-04-2015.

34. Lijst van vragen en antwoorden. **Jaarverslag en slotwet Ministerie van Volksgezondheid, Welzijn en Sport 2014**  17 Juni 2015.

35. **Jaarverslag en slotwet Ministerie van Volksgezondheid, Welzijn en Sport 2015**  8-05-2016.

36. Lijst van vragen en antwoorden. **Jaarverslag en slotwet Ministerie van Volksgezondheid, Welzijn en Sport 2015**  17-06-2016.

37. Nieuwsbericht. **Kabinet investeert fors in verpleeghuiszorg**  15-09-2015.

38. Nieuwsbericht. **Kwaliteit van de zorg sterk verbeterd, zorguitgaven gestabiliseerd**  16-09-2014.

39. **Lijst van vragen en antwoorden over het Nederlandse Stabiliteitsprogramma2015** 21-04-2015.

40. **Miljoenennota 2015**  16-09-2014.

41. Rijksoverheid. **Miljoenennota 2016**  15-09-2015.

42. **Miljoenennota internetbijlagen**

43. Rijksoverheid. **Ministerie van Volksgezondheid, Welzijn en Sport (XVI) Rapport bij het jaarverslag**  Mei 2016.

44. Directie Begrotingszaken. **Najaarsnota 2014**  21 november 2014.

45. Ministerie van financien. **Najaarsnota 2015**  27-11-2015.

46. Raad van State. **No.W06.14.0284/III/B**  11-09-2014.

47. Raad van State. **No.W06.16.0070/III/B**  8-04-2016.

48. **Nota over de toestand van ’s rijks financiën**  16-09-2014.

49. **Nota over de toestand van ’s rijks financiën**  15-09-2015.

50. Rijksoverheid. **Ontwerpbegrotingsplan Nederland**  September 2015.

51. rijksoverheid. **Ontwerpbegrotingsplan Nederland**  September 2014.

52. **Parlementaire enquête Woningcorporaties**  21 juni 2014.

53. CBS. **Persbericht CBS Tekort overheid 2014 met 2,3 procent gelijk aan jaar eerder**  26-03-2015.

54. Rijksoverheid. **Rapport Studiegroep Duurzame Groei**  Juli 2016.

55. rijksoverheid. **Rapport werkgroep Zorg ten behoeve van de Studiegroep Duurzame Groei**  Juli 2016.

56. Welzijn en Sport Ministerie van Volksgezondheid. **Rijksjaarverslag 2014 xvi Volksgezondheid, Welzijn en Sport**  20-05-2015.

57. rijksoverheid. **Rijksjaarverslag 2015 xvi Volksgezondheid, Welzijn en Sport**  18-05-2016.

58. Algemene rekenkamer. **Staat van de rijksverantwoording 2015 Rijksbrede resultaten verantwoordingsonderzoek**  2016.

59. **Taakopdracht Instellen Technische werkgroep Beheersinstrumentarium Zorguitgaven**

60. Algemene rekenkamer. **Trendrapport open data 2016**  24-03-2016.

61. TNO. **Trendrapport Bewegen en Gezondheid 2000/2014**  2015.

62. Verslag houdende een lijst van vragen en antwoorden. **Vaststelling van de begrotingsstaten van het Ministerie van Volksgezondheid, Welzijn en Sport (XVI) voor het jaar 2015**  13-11-2014.

63. Welzijn en Sport Ministerie van Volksgezondheid. **Vaststelling van de begrotingsstaten van het Ministerie van Volksgezondheid, Welzijn en Sport (XVI) voor het jaar 2015**  16-09-2014.

64. Memorie van toelichting. **Vaststelling van de begrotingsstaten van het Ministerie van Volksgezondheid, Welzijn en Sport (XVI) voor het jaar 2015**  Unknown.

65. **Verslag van een wetgevingsoverleg**  23-06-2016.

66. Raad van State. **Voorjaarsrapportage Begrotingstoezicht 2015**  13-04-2015.

67. Raad van State. **Voorjaarsrapportage Begrotingstoezicht 2015**  13-04-2015.

68. Minister van Volksgezondheid Welzijn en Sport. **Vragen gesteld door de leden der Kamer, met de daarop door de regering gegeven antwoorden over het bericht dat het eigen risico niet meer te betalen is**  3-02-2015.

69. Minister van Volksgezondheid Welzijn en Sport. **Vragen gesteld door de leden der Kamer, met de daarop door de regering gegeven antwoorden over hoe de kosten van het huidige zorgstelsel ongezien naar de burger verschoven** 11-02-2016.

70. Verslag van een schriftelijk overleg. **Waardering van risico’s bij publieke investeringsprojecten**  21-04-2016.

71. RIVM. **Zorgbalans 2014 De prestaties van de Nederlandse gezondheidszorg**  2014.

72. RIVM. **Zorgbalans 2014 Op hoofdlijnen**  2014.

**Costs of smoking**

1. Nuchter Kenniscentrum Leeftijdsgrenzen. **Alcohol& tabaksverkoop aan jongeren 2016**  2016.

2. Technische werkgroup zorgkeuzes in kaart. **Analyse van beleidsopties voor de zorg van tien politieke partijen>**  Maart 2015.

3. Technische werkgroep Zorgkeuzes in Kaart. **Beantwoording feitelijke vragen door de Technische werkgroep Zorgkeuzes in Kaart**  17-04-2015.

4. WHO. **Global status report on alcohol and health**  2011.

5. HaskoningDHV Nederland B.V. **Risico's in perspectief Risicovergelijking**  November 2014.

**Bedsores**

1. **Bijlage 2 Waardig leven met zorg Overzicht van initiatieven gericht op vernieuwing van de langdurige zorg**
2. Staatssecretaris van Volksgezondheid Welzijn en Sport. **Brief van de staatssecretaris van volksgezondheid, welzijn en sport over Langdurige zorg**  26-02-2016.
3. **Concept verslag Wet langdurige zorg**  11-09-2014.
4. Stichting KOH Meetpunt Kwaliteit. **De verpleegkundig specialist in de eerste lijn. Eindrapport.**  Februari 2015.
5. Maastricht University. **Landelijke Prevalentiemeting Zorgproblemen Rapportage resultaten 2013**  2013.
6. Gewijzigd amendement van het lid agema ter vervanging van dat gedrukt onder nr. 101. **Regels inzake de verzekering van zorg aan mensen die zijn aangewezen op langdurige zorg (Wet langdurige zorg)**  15-09-2014.
7. RIVM. **Zorgbalans 2014**  2014.

**Hospital mortality**

1. Tweede Kamer. **Agenda Procedurevergadering**  22-01-2015.
2. NZA. **Beleidsbrief Marktscan medisch specialistische zorg 2014**  4-12-2014.
3. Verslag van een schriftelijk overleg. **Beleidsdoelstellingen op het gebied van Volksgezondheid, Welzijn en Sport**  22-06-2016.
4. Ministerie van Volksgezondheid Welzijn en Sport. **Betreft Commissiebrief Tweede Kamer inzake VSO inzake Resultaten van het Jaar van de transparantie (32620, nr.168)**  22-06-2016.
5. Minister van volksgezondheid welzijn en sport. **Brief initiatiefnota van de leden Anne Mulder en Michiel van Veen over patiëntveiligheid**  29-01-2014.
6. Minister van Volksgezondheid Welzijn en Sport. **Brief Invoering Diagnose Behandeling Combinaties (DBCs)**  16-12-2014.
7. Minister van Volksgezondheid Welzijn en Sport. **Brief Kwaliteit van zorg**  18-07-2014.
8. NIVEL CBS, NJi, NZA, RIVM, SCP, Trimbos instituut, ZIN. **De Staat van Volksgezondheid en Zorg Kerncijfers voor beleid - een introductie -**  Mei 2016.
9. IGZ. **Het resultaat telt ziekenhuizen 2013**  Februari 2015.
10. IGZ. **Het resultaat telt ziekenhuizen 2014**  Januari 2016
11. Ministerie van Volksgezondheid Welzijn en Sport. **Kamerbrief Transparantie van kwaliteit in de curatieve zorg**  18-07-2014.
12. Verslag van een schriftelijk overleg. **Kwaliteitszorg**  10-10- 2015.
13. NZA. **Marktscan en beleidsbrief Medisch specialistische zorg 2014**  December 2014.
14. IGZ. **Meer oog voor kwaliteit bij cardiothoracaal chirurgische centra**  April 2015.
15. ZI. **Rapport Ontwikkeling algemene indicatoren**  Januari 2016.
16. **Resultaten Rijksbegroting 2014**
17. Welzijn en Sport Ministerie van Volksgezondheid. **Rijksbegroting 2015 xvi Volksgezondheid, Welzijn en Sport**  16-09-2014.
18. Welzijn en Sport Ministerie van Volksgezondheid. **Rijksjaarverslag 2014 xvi Volksgezondheid, Welzijn en Sport** 20-05-2015.
19. Memorie van Toelichting. **Vaststelling van de begrotingsstaten van het Ministerie van Volksgezondheid, Welzijn en Sport (XVI) voor het jaar 2015**  2014.
20. Minister van Volksgezondheid Welzijn en Sport. **Vragen gesteld door de leden der Kamer, met de daarop door de regering gegeven antwoorden over het bericht dat het Nederlands ziekenhuis in het weekend**  2-09-2015.
21. RIVM. **Zorgbalans 2014**  September 2014.
